# Childhood Cancer Awareness Program in Bungoma County, Kenya

**DOI:** 10.1007/s13187-024-02468-z

**Published:** 2024-06-22

**Authors:** Larissa Klootwijk, Lilian Apadet Osamong, Sandra Langat, Festus Njuguna, Sally Kimaiyo, Terry A. Vik, Gertjan Kaspers, Saskia Mostert

**Affiliations:** 1 Princess Máxima Center for Pediatric Oncology, Utrecht, The Netherlands; 2 Emma Children’s Hospital, Amsterdam UMC, Vrije Universiteit, Amsterdam, The Netherlands; 3 Academic Model Providing Access to Healthcare (AMPATH), Eldoret, Kenya; 4 Department of Child Health and Pediatrics, Moi Teaching and Referral Hospital, Moi University, Eldoret, Kenya; 5 Department of Pediatrics, Indiana University School of Medicine, Indianapolis, USA

**Keywords:** Low- and middle-income countries, Primary healthcare, Childhood cancer, Training, Awareness

## Abstract

**Background:**

Awareness could play a key role in reducing underdiagnosis and accelerating referral of childhood cancer in low- and middle-income countries and ultimately improve outcomes. This study describes the implementation of a childhood cancer awareness program in Bungoma County in Kenya, containing five components: (1) baseline data collection of primary healthcare facilities; (2) live training session for healthcare providers (HCP); (3) early warning signs posters; (4) online SMS course for HCP; and (5) radio campaign.

**Methods:**

This study was conducted between January and June 2023. All 144 primary healthcare facilities (level 2 and 3 health facilities) within Bungoma County were visited by the field team.

**Results:**

All 125 level 2 (87%) and 19 level 3 (13%) facilities participated in the study. National Health Insurance Fund (NHIF) failed to cover services in 37 (26%) facilities. HCP were more often reported absent at level 3 (89%) than level 2 (64%) facilities (*P* = 0.034). The 144 live training sessions were attended by over 2000 HCP. Distribution of 144 early warning signs posters resulted in 50 phone calls about suspected childhood cancer cases. Sixteen children were later confirmed with childhood cancer and treated. Online SMS learning was completed by 890 HCP. Knowledge mean scores improved between pre-test (7.1) and post-test (8.1; *P* < 0.001). Finally, 540 radio messages about childhood cancer and a live question-and-answer session were broadcasted.

**Conclusion:**

This study described the implementation of a childhood cancer awareness program in Kenya involving both HCP and the general public. The program improved HCP’s knowledge and increased the number of referrals for children with cancer.

**Supplementary Information:**

The online version contains supplementary material available at 10.1007/s13187-024-02468-z.

## Introduction

Underdiagnosis remains one of the biggest challenges when addressing childhood cancer outcomes in low- and middle-income countries (LMIC) [1]. In Kenya, for example, only 10–20% of the estimated 5000 children that annually develop cancer are diagnosed and treated in a referral hospital [2, 3]. The WHO Global Initiative for Childhood Cancer aims for survival rates of 60% for children with one of six common and curable types of cancers [4]. However, as long as only a minority of children with cancer are diagnosed, this goal cannot be achieved.

Numerous reasons exist why many children are never diagnosed with cancer or receive their diagnosis extremely late. This problem is driven by both patient-related and healthcare provider (HCP)-related factors. Low health literacy in rural communities in LMIC poses a challenge for parents to identify symptoms and medical needs accurately. Many opt to seek traditional care instead of reaching out to conventional health facilities due to alternative beliefs [5, 6]. Parents may not have money to pay for care services [7]. Moreover, when children are seen in primary care facilities, HCP often fail to recognize childhood cancer, as the disease is rare and resembles symptoms from common illnesses such as malaria, meningitis, or tuberculosis [8]. Additionally, once HCP suspect childhood cancer, immediate referral to the hospital that provides comprehensive childhood cancer care can be difficult [9–11]. To limit these delays and ensure timely referral, studies highlight need for childhood cancer awareness campaigns among HCP and communities [12]. Although awareness campaigns could be a key tool to reduce underdiagnosis, few have been implemented [12].

This study describes the implementation of a childhood cancer awareness program in Bungoma County in Kenya. The program contains five components: (1) baseline data collection of primary health facilities; (2) live training sessions of HCPs; (3) posters with early warning signs; (4) online short message service (SMS) course for HCPs; and (5) radio campaign.

## Methods

### Setting

Kenya is a LMIC in East Africa, counting a total population of 53 million people. Kenya has 47 counties which each have a County Ministry of Health and Sanitation Department that supervises health facilities within the county [13]. Bungoma county is located in the western Kenya, hosting approximately 1.8 million people of which 680,000 are below 18 years [14]. Annually, 140 children are expected to develop cancer in this region (estimated prevalence: 0.002%) [15]. However, a retrospective analysis showed that currently only 13 children from Bungoma are diagnosed and treated for childhood cancer at Moi Teaching and Referral Hospital (MTRH) per year and their survival rate is low [16].

**Fig. 1:**
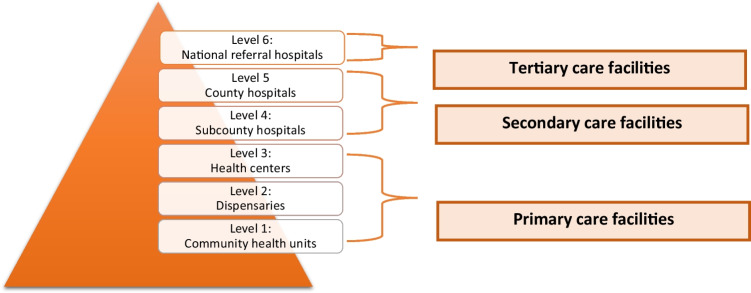
Six levels of health facilities in Kenya

The Kenyan healthcare system consists of six healthcare levels [17] (Fig. 1). Healthcare services are mostly provided by level 2 and 3 facilities. These are basic health facilities that offer childhood immunizations and treatment for common childhood illnesses, malaria, and HIV. The vast majority of Bungoma’s population (90%) resides in rural areas and depends on these level 2 and 3 facilities [18]. Bungoma has 125 level 2 and 19 level 3 facilities. Level 2 facilities (dispensaries) are usually managed by nurses. Each level 2 facility is connected to one or more community health extension workers supervising 10–30 community health volunteers (depending on the catchment area of the facility). These volunteers have received short training on common illnesses and play a crucial role in educating communities and referring to dispensaries. Level 3 facilities (health centers) are usually staffed by clinical officers, who completed 3 years of training, and several nurses.

Patients from Bungoma who need referral from level 2 and 3 facilities are usually referred to either sub-county hospital (level 4 facility), county hospital (level 5 facility), or MTRH, the national referral hospital (level 6 facility) for western Kenya as per the standard of care [19, 20]. MTRH is the only public hospital in western Kenya that offers comprehensive childhood cancer treatment [21]. The pediatric oncology unit is supervised by two pediatric oncologists. Treatment modalities include chemotherapy, surgery, and radiotherapy [22]. National Health Insurance Fund (NHIF) is the national health insurance system of Kenya and covers most costs for childhood cancer treatment. Sub-packages of NHIF include “Linda Mama” and “Edu Afya.” Linda Mama covers maternal care and children under 5 years old. Edu Afya covers children in high school [23].

### Study Design

**Fig. 2:**
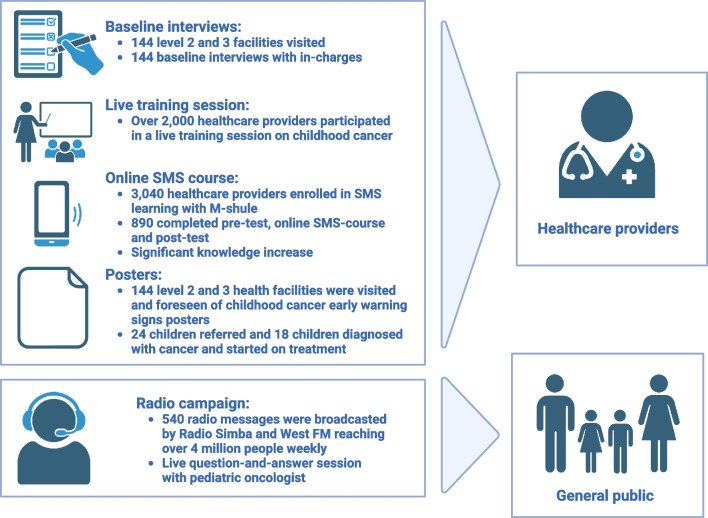
The five components of the childhood cancer awareness program

This prospective study described the implementation of a childhood cancer awareness program in Bungoma. Figure 2 illustrates its five components: (1) baseline data collection of primary health facilities; (2) live training session of HCP; (3) posters with early warning signs of childhood cancer; (4) online SMS course for HCP; and (5) radio campaign. This multiple component strategy was used to reach both HCP and the general public. This is necessary as the causes of delays in access to childhood cancer care are rooted in these groups. As primary healthcare facilities have very limited resources in Kenya, the purpose of the program was not for primary staff members to diagnose children with cancer but solely to recognize children with cancer and refer them appropriately.

Between January and June 2023, the field team visited all level 2 and 3 health facilities within Bungoma. This field team consisted of one researcher and one research nurse. Prior to the field team visit, the public health officer that supervised these level 2 and 3 facilities, informed in-charges of each level 2 or 3 facility about the date of the visit of the field team for the training. Public health officers are HCP with a diploma in public health.

#### Baseline Data Collection of Primary Health Facilities

Interviews with in-charges of all level 2 and 3 facilities on baseline facility information were conducted by the research nurse using a semi-structured questionnaire. This questionnaire was designed by a panel of Kenyan, American, and Ductch doctors. A pilot study was executed with 20 healthcare facilities and minor revisions were made to the questionnaire after this pilot. Informed consent from in-charges was obtained before the interview. Data collected included number and type of permanent staff, available laboratory services, NHIF accreditation status, referral pathways in use, and staff absent on duty regardless of the reason. Absenteeism is defined as a HCP who is hired at a facility but is not present on duty at that particular day of the visit [24].

#### Live Training Session on Childhood Cancer

At each facility, permanent-, support-, and affiliated staff were invited to join the live training session. The live training session consisted of a PowerPoint presentation provided by the researcher. Staff were taught about the epidemiology of childhood cancer globally, and in Kenya and Bungoma specifically. Staff were trained on early signs and symptoms of different common and curable types of cancers as defined by the WHO criteria: acute lymphoblastic leukemia, Burkitt lymphoma, Hodgkin lymphoma, retinoblastoma, nephroblastoma, and low-grade glioma. Moreover, sessions covered importance of NHIF registration and which referral pathways to use. HCP attending the session received 500 Kenyan Shillings as a travel reimbursement.

#### Posters with Early Warning Signs of Childhood Cancer

**Fig. 3:**
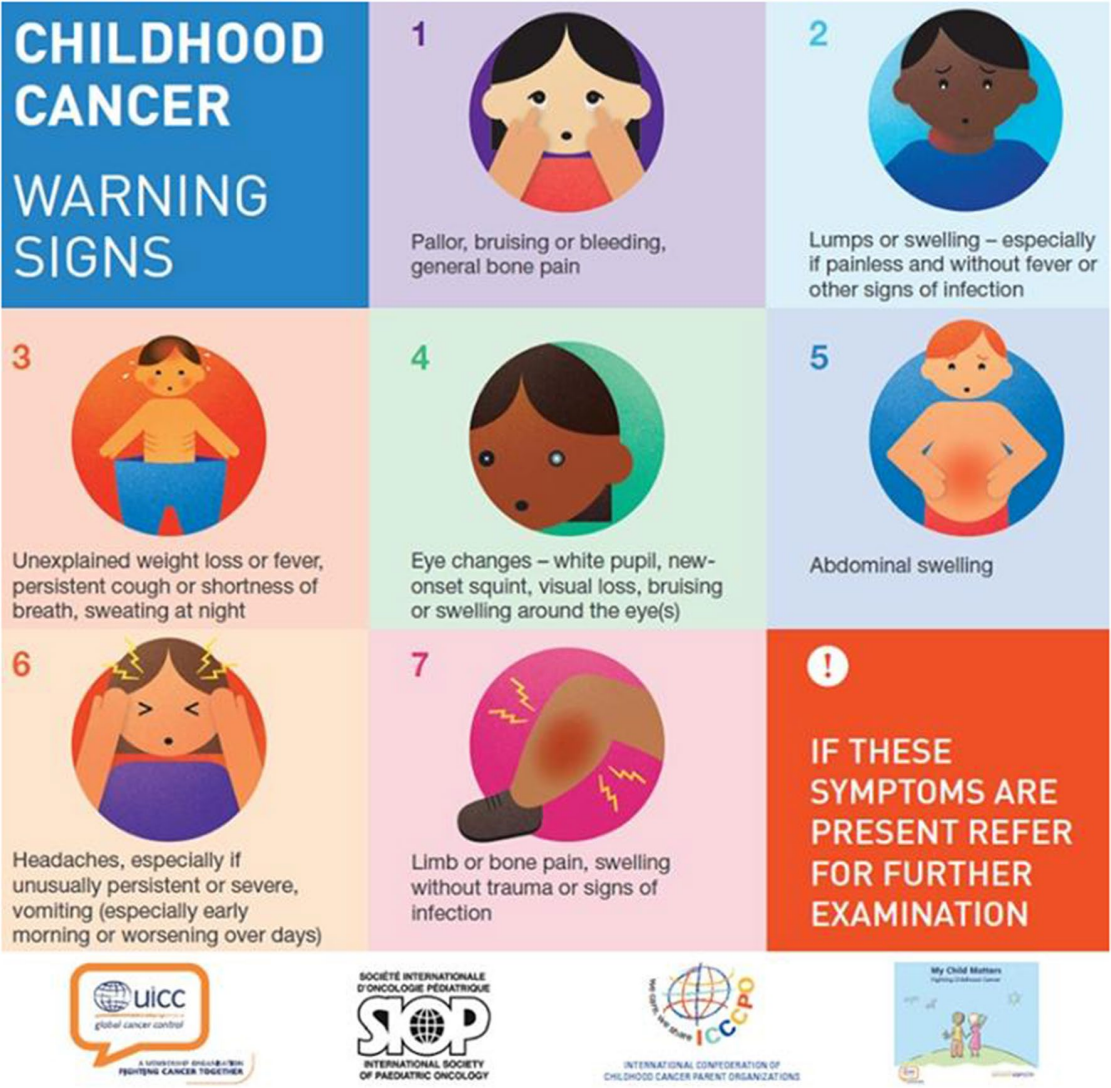
SIOP early warning signs poster

The field team explained the SIOP early warning signs poster to the participants (Fig. 3) [25]. This poster emphasizes six common and curable types of cancers and osteosarcoma. The field team subsequently affixed the poster on a visible location in the facility together with the in-charge. Contact numbers of the pediatric oncology team at MTRH were indicated on the poster for communication on referral of any suspected cases. Impact of the poster was measured in terms of phone calls to the team using the provided phone numbers.

#### Online SMS Course About Childhood Cancer

A digital learning strategy was implemented using SMS messages through the Kenyan M-shule platform [26]. This course enabled HCP to learn about childhood cancer in their own time through SMS. The course consisted of 7 learning modules. Module 1 contained an introduction to childhood cancer, importance of NHIF, and adequate referral pathways. Subsequently, modules 2 through 7 addressed signs, symptoms, and treatment options of the six common and curable types of cancer. All interested HCP present at the live session were enrolled after verbal consent. The field team provided the M-shule platform with a list of consented participants weekly. HCP who could not attend the live training session were registered for the SMS course as well. Participants were usually able to start learning within 7 days after the live training session. The content for the SMS learning and the questions were developed in collaboration with the pediatric oncology team at MTRH and the experts from M-shule. Impact of the SMS learning was measured by analyzing the knowledge increase of the HCP on childhood cancer.

Knowledge increase was measured through the pre-test and post-test. Before HCP started the online SMS course, a pre-test of 11 questions was conducted through SMS to establish the knowledge level on childhood cancer (Table 2). At the end of the online SMS course, a post-test with the same eleven questions was conducted. Grading was done by attributing one point per correctly answered question. In total, one could obtain 11 points maximum. Upon completing the online SMS course, HCP received a certificate.

#### Radio Campaign

A radio campaign was conducted to raise awareness about childhood cancer among communities in Bungoma. In Kenya, access to a radio at home is very high for both rural (95%) and urban (94%) populations [27]. Two radio stations broadcasted messages in Kiswahili and Bukusu on childhood cancer daily for 6 months. The radio messages contained three topics: (a) basic information on what childhood cancer is; (b) when to think of childhood cancer and what to do when you suspect cancer; and (c) NHIF coverage (Supplementary File 1). In addition, a live question-and-answer session was conducted once by a pediatric oncology fellow from MTRH in the radio studio.

### Data Analysis

All data were collected on an Android tablet using the application of Castor version 0.1.45. After finalizing data collection, data were transferred to Excel. Data management and analysis were performed using SPSS version 26.0. Frequency distributions, medians, means, and standard deviations were calculated for baseline data. Univariate analysis was used to assess results of the online SMS course. Influence of sex, staff or facility types on outcome of pre-test and post-test was measured with chi-squared test and paired *t*-testing. A two-tailed *p*-value < 0.05 was considered statistically significant.

## Results

Between January and June 2023, all 144 level 2 and 3 health facilities were visited and participated in the study (response rate 100%).

### Baseline Data Collection of Primary Health Facilities

Table 1 presents results of baseline interviews with HCP. All 144 in-charges (100%) of level 2 and 3 health facilities agreed to be interviewed. Their professions were clinical officers (11%) and nurses (89%). Table 1 illustrates that the 144 facilities included 125 level 2 (87%) and 19 level 3 (13%) facilities. Level 2 facilities were generally staffed by at least one nurse and sometimes a lab technician (59%). Clinical officers were only employed at level 2 facilities in 15% of facilities. Level 3 facilities had at least one clinical officer, nurse, and lab technician. Laboratory facilities were not available at any primary healthcare facility that was visited. In-charges solely reported availability of point-of-care hemoglobin testing using Hemocue (67%) and microscopy (71%) to diagnose malaria. In total, 37 facilities (26%) were not accredited to offer NHIF coverage, others offered either full package (14%) or sub-package (60%). Some in-charges of facilities that did not offer full NHIF packages indicated that they had applied for NHIF but that activation in the facility remained challenging. Others specified that they did not meet NHIF facility accreditation criteria. In-charges reported using the following patient referral pathways: level 4 (50%), level 5 (45%), level 6 (0%), or to another facility (5%).

**Table 1:** Baseline data of primary health facilities (*n* = 144)

	Total	Level 2 health facility	Level 3 health facility
Facilities	144 (100%)	125 (87%)	19 (13%)
Permanent staff per facility			
Clinical officers (*n* ≥ 1)	38 (26%)	19 (15%)	19 (100%)
Nurses (*n* ≥ 1)	144 (100%)	125 (100%)	19 (100%)
Lab technicians (*n* ≥ 1)	93 (65%)	74 (59%)	19 (100%)
Laboratory services per facility			
Hemocue	97 (67%)	80 (64%)	17 (90%)
Microscopy	103 (72%)	84 (67%)	19 (100%)
NHIF accreditation per facility			
Full package	20 (14%)	12 (10%)	8 (42%)
Only Linda Mama and Edu Afya*	61 (42%)	56 (45%)	5 (26%)
Only Linda Mama	26 (18%)	23 (18%)	3 (16%)
None	37 (26%)	34 (27%)	3 (16%)
Used referral pathways per facility			
Level 4 (subcounty hospital)	73 (51%)	66 (53%)	7 (37%)
Level 5 (county hospital)	63 (44%)	52 (42%)	11 (58%)
Level 6 (national referral hospital)	0 (0%)	0 (0%)	0 (0%)
Other hospital (e.g., level 2/3/private)	8 (6%)	7 (6%)	1 (5%)

^*^Linda Mama NHIF program only covers maternal care and care for children under 5. Edu Afya NHIF program covers for children in secondary schools

Staff was more often found absent on the day of the visit, at level 3 (89%) than at level 2 (64%) facilities (*P* = 0.034). Relatively, more clinical officers were absent at level 3 (*n* = 11, 58%) than at level 2 (*n* = 6, 32%) facilities (*P* < 0.001). Relatively, more nurses were absent at level 3 (*n* = 17, 89%) than at level 2 (*n* = 72, 58%) facilities (*P* = 0.034). Relatively, more lab technicians were absent at level 3 (*n* = 9, 47%) than at level 2 (*n* = 17, 26%) facilities (*P* = 0.024).

### Live Training Session on Childhood Cancer

The live training sessions at 144 level 2 and 3 health facilities were attended by over 2000 HCP: permanent staff (in-charges, clinical officers, nurses, lab technicians), support staff (clerks, casuals, mentor mothers, patient educators, students, health records/information officers, HIV-testing services officers, nutritionists), and affiliated staff (public health officers, community health extension workers, community health volunteers). The live session took between 30 and 60 min. After the live session, there was time to ask questions and discuss any potential cases from the communities. Questions asked included clarification on referral process and financial reimbursement for travel expenses of suspected childhood cancer cases from and to MTRH. Children suspected of cancer referred through the program received a reimbursement for the travel cost to reduce delays in referral due to financial constraints. Participants shared their experiences with white decolorization of the eye in potential retinoblastoma cases.

### Posters with Early Warning Signs of Childhood Cancer

At all 144 facilities, the field team explained the SIOP early warning signs poster to participants after the live training session. During the first year after launching the awareness program, the pediatric oncology team at MTRH received approximately 50 calls (using the phone number on the poster) about suspected childhood cancer cases. Twenty-four of these cases were referred to MTRH of which 16 were confirmed to have childhood cancer and started on treatment: acute lymphoblastic leukemia (*n* = 3), nephroblastoma (*n* = 3), Burkitt lymphoma (*n* = 2), brain tumor (*n* = 2), Hodgkin lymphoma (*n* = 2), rhabdomyosarcoma (*n* = 2), yolk sac tumor (*n* = 1), acute myeloid leukemia (*n* = 1), nasopharyngeal carcinoma (*n* = 1). In total, 4 of these 16 children have passed away (25%).

### Online SMS Course About Childhood Cancer

In total, 3040 HCP were enrolled on the SMS-learning platform. In total, 890 (29%) HCP completed the full program, 1497 HCP (49%) did not complete the full program due to technical issues, and 653 HCP (21%) were excluded as they did not complete the full program in chronological order: pre-test, online SMS course, post-test. The technical challenges included blocked spam messages, causing HCP not to receive SMS-learning messages, or full inbox, leading to HCP not receiving new messages. SMS course took 30–120 min. Table 2 presents pre-test and post-test questions with summary answers provided by all 890 participants. Pre- and post-test took 5–20 min. Questions about retinoblastoma, Wilms tumor, and Hodgkin lymphoma were most often incorrectly answered.

**Table 2:** Results of online SMS-course: pre-test and post-test of participants (*n* = 890)

Question	Answer	Pre-test all healthcare providers	Post-test all healthcare providers
*Cancer is:*	A. **A non-communicable disease**	**700 (79%)**	**724 (81%)**
	B. A communicable disease	190 (21%)	166 (19%)
*The major groups of cancers are blood cancers, solid cancers, lymphomas and brain tumors*	A.** Agree**	**831 (93%**	**841 (95%)**
	B. Disagree	59 (7%)	49 (5%)
*Approximately how many children develop cancer per year in Kenya*	A. 1,000,000	212 (24%)	245 (28%)
	B. 50	142 (16%)	154 (17%)
	C. **3000**	**536 (60%)**	**491 (55%)**
*Cancer is treatable with chemotherapy (drugs) and surgery*	A.** Agree**	**854 (96%)**	**869 (98%)**
	B. Disagree	36 (4%)	21 (2%)
*When do you have to think of blood cancers?*	A. Persistent hotness of the body	39 (4%)	37 (4%)
	B. Pallor	124 (14%)	97 (11%)
	C. Bleeding and bruises	174 (20%)	180 (20%)
	D. **All the above**	**553 (62%)**	**576 (65%)**
*Which childhood cancer commonly presents with fever, bleeding, anemia, enlarged lymph nodes, and hepatosplenomegaly?*	A.** Acute lymphoblastic leukaemia**	**753 (85%)**	**759 (85%)**
	B. Wilms tumor	71 (8%)	72 (8%)
	C. Brain tumor	66 (7%)	59 (7%)
*Which cancer can be hereditary?*	A. Acute lymphatic leukemia	383 (43%)	368 (41%)
	B. Burkitt lymphoma	106 (12%)	114 (13%)
	C. Hodgkin lymphoma	75 (8%)	49 (6%)
	D. **Retinoblastoma**	**326 (37%)**	**359 (40%)**
*Which is the commonest childhood kidney cancer*	A. Hodgkin lymphoma	238 (27%)	209 (24%)
	**B. Wilms tumor**	**460 (52%)**	**524 (59%)**
	C. Burkitt lymphoma	192 (22%)	157 (18%)
*A child who presents with severe headache, convulsions, projectile vomiting, and abnormal gait would most likely be suffering from which type cancer*	A. Wilms tumor	86 (10%)	78 (9%)
	B.** Brain tumor**	**727 (82%)**	**735 (83%)**
	C. Hodgkin lymphoma	77 (9%)	77 (9%)
*Z.A. who is 14 years presents with a 1-year history of enlarged cervical lymph nodes. He has fever, night sweats, and weight loss, which childhood cancer is he likely to be suffering from*	A. Acute lymphoblastic leukaemia	361 (41%)	336 (38%)
	B. Burkitt lymphoma	264 (30%)	2237 (27%)
	C. **Hodgkin lymphoma**	**265 (30%)**	**317 (36%)**
*Brain tumors can present with convulsion, morning headache and vomiting, and problems with walking*	A.** Agree**	**841 (95%)**	**849 (95%)**
	B. Disagree	49 (6%)	41 (5%)

Table 3 presents total test scores of different types of participants and facilities. When comparing pre-test and post-test scores, the mean score for all HCP in pre-test is 7.1 (SD 1.6) and in post-test 8.1 (SD 1.5). Paired *T*-test shows a statistically significant difference for pre- and post-test of *P* < 0.001 (95% CI 0.02–0.28). The type of staff significantly influenced scores on both pre-test and post-test but did not significantly influence improvement. The facility level did not statistically significantly influence test scores.

**Table 3:** Results of online SMS-course: pre-test and post-test per socio-demographic characteristics of participants (*n* = 890)

Socio-demographic	No. participants	Pre-test result				Post-test result				Improvement			
characteristics:	(%)	Mean (SD)	Median	Range	*P*	Mean (SD)	Median	Range	*P*	Mean (SD)	Median	Range	*P*
All participants	890 (100%)	7.1 (1.6)	7.0	1.0–10.0	-	8.1 (1.5)	8.0	2.0–10.0	-	1.1 (1.7)	1.0	− 5–7.0	-
Gender													
Female	548 (62%)	7.0 (1.6)	7.0	1.0–10.0	0.699	8.1 (1.5)	8.0	2.0–10.0	0.043	1.1 (1.8)	1.0	− 5.0–7.0	0.176
Male	275 (31%)	7.2 (1.7)	7.0	2.0–10.0		8.3 (1.5)	9.0	5.0–10.0		1.1 (1.6)	1.0	− 4.0–7.0	
Type of staff													
Nurse	39 (4%)	8.1 (1.3)	8.0	5.0–10.0	< 0.001	9.1(0.8)	9.00	8.0–10.0	< 0.001	1.0 (0.6)	1.0	− 2.0–5.0	0.061
Community health volunteer	765 (86%)	6.9 (1.6)	7.0	1.0–11.0		8.0(1.5)	8.00	2.0–10.0		1.1 (1.7)	0.0	− 5.0–7.0	
Community health extension worker	33 (4%)	7.7 (1.3)	8.0	5.0–10.0		9.0 (1.1)	9.00	6.0–10.0		1.2 (1.4)	1.0	− 1.0–3.0	
Public health officer	11 (1%)	8.4 (1.4)	9.0	6.0–11.0		9.5 (0.9)	10.0	7.0–10.0		1.3 (0.8)	1.5	0–2.0	
Clinical officer	6 (0.5%)	8.1 (0.4)	8.0	8.0–9.0		9.5 (0.5)	9.5	9.0–10.0		0.8 (1.0)	0.5	0.0–2.0	
Lab technician	4 (0.5%)	8.5 (0.6)	8.5	8.0–9.0		9.3 (0.5)	9.0	9.0–10.0		− 1 (1.00)	1.0	0.0–2.0	
Support staff*	32 (4%)	7.5 (1.3)	8.0	5.0–10.0		8.4 (1.3)	9.0	6.0–10.0		0.9 (1.3)	1.0	− 2.0–4.0	
Type of facility													
Level 2	774 (87%)	7.1 (1.6)	7.0	1.0–10.0	0.312	8.2 (1.5)	8.0	2.0–10.0	0.823	1.1 (1.7)	1.0	− 5.0–7.0	0.777
Level 3	116 (13%)	6.9 (1.5)	7.0	3.0–10.0		8.1(1.5)	8.0	4.0–10.0		1.1 (1.6)	1.0	− 2.0–5.0	

^*^Support staff included: clerks, casuals, mentor mothers, patient educators, students, health records and information officers, HIV-testing services officers, and nutritionists

### Radio Campaign

In total, 540 radio spots about childhood cancer were broadcasted by the contracted radio stations: Radio Simba and Radio West FM. These radio messages covering three main topics were broadcasted twice daily during the most listened hours: in the morning between 6 and 8 AM before going to the farms or work, and in the evening between 6 and 8 PM when returning home. During the live question-and-answer session, people could call the station with their questions which were then answered on the spot. In total, ten phone calls were taken during the show. Questions included topics such as the following: “What are the causes of childhood cancer? What is the danger of childhood cancer? How can one best handle childhood cancer? Where can a child be examined for cancer? Which stages of cancer are there? Can children get cancer of the head and neck? Can children acquire cancer through sharing food or blood transfusions?”.

Some HCP who joined the live session of the awareness program confirmed that they had heard the radio messages. Considering the large audience of both radio stations in western Kenya (Radio Simba: 3.5 million listeners and Radio West FM: 2.8 million listeners), most Bungoma county residents are expected to listen to these two channels.

## Discussion

This study describes the implementation of a blended strategy using both digital and non-digital components to raise awareness on childhood cancer amongst HCP and the general public. The five components of the childhood cancer awareness program in Bungoma County included (1) baseline data collection of primary health-care facilities, (2) live training session of HCP, (3) posters with early warning signs, (4) online SMS course for HCP, and (5) radio campaign. This study showed limited availability of full NHIF package and laboratory services at level 2 and 3 facilities. High numbers of staff were not present on duty, which might threaten access to healthcare services. In total, 144 live training sessions and 144 posters with early warning signs were provided. SMS learning was completed by 890 participants (29%). The uptake was relatively low due to profound technical problems, which illustrates the need for prompt anticipation and mitigation. The SMS course resulted in a significant improvement in HCP’s knowledge of childhood cancer. Finally, 540 radio messages were sent out to communities. One year after launching the program, 16 children with confirmed cancer have been referred from Bungoma County to MTRH for treatment. This program thus illustrates that it is possible to increase childhood cancer awareness among primary healthcare workers, who are the backbone in prevention and early detection of many diseases.

The limited availability of NHIF accredited primary healthcare facilities could negatively impact children with cancer. Health insurance is shown to benefit childhood cancer outcomes in HIC and LMIC [28, 29]. A large cohort of children with cancer at MTRH showed survival was indeed significantly better in those with health insurance [29, 30]. Many Kenyans however fail to afford NHIF due to poverty [31]. Additionally, considering that the majority of level 2 and 3 facilities are not NHIF accredited, it stands to reason that families only start to register when referred for more specialized care to level 4, 5, or 6 facilities. These findings emphasize the importance of ensuring NHIF accreditation in primary care facilities for families to benefit from these healthcare services. Moreover, these findings reveal social inequalities in the affordability and availability of NHIF that warrant advocacy for health-insurance coverage for childhood cancer in Kenya.

Absence of staff on duty was observed in the majority of visited level 2 and 3 facilities. Two types of absenteeism can be distinguished: involuntary and voluntary. Involuntary absence on duty includes absence related to legitimate reasons for absenteeism, whereas voluntary absence does not. Our study was not designed to differentiate between the two types of absenteeism; however, the high absence rate measured could have been related to both involuntary and voluntary reasons [32, 33]. HCP could have been on leave, needed to go for training elsewhere, or were simply not on call. Yet, it could also refer to power abuse, financial mismanagement, low salaries, lack of motivation, and dysfunctional governance structures. Absenteeism in LMIC is reported to average between 25 and 40% in LMIC, and can pose a serious threat to availability of healthcare services and universal health coverage [34–36]. Our findings suggest that further analysis of absenteeism in Kenya could be useful.

The live session and the posters were appreciated by the HCP. The number of phone calls to the pediatric oncology team at MTRH suggests a good response to the awareness campaign. The direct referral of 24 children from Bungoma since the launch, of whom 16 now receive cancer treatment, already surpasses the normal annual average by 23%. Prior to the program, in-charges stated they would never refer to MTRH directly, although studies have shown that direct referral to a childhood cancer care facility increases survival outcomes [9]. A systematic review on the effectiveness of childhood cancer training sessions in other LMIC showed similar positive results [12]. The majority of these trainings resulted in a knowledge gain of attendees [37–40]. Another study reporting on live childhood cancer training to HCP of level 5 facilities in Kenya also reported increased knowledge [41, 42]. Long-term follow-up focusing on detailed analysis of the referrals is however needed to evaluate the impact of our campaign properly.

The online SMS course demonstrated a significant knowledge gain among participants. However, its uptake faced profound technical challenges that need to be addressed. Interestingly, we found significant differences in test results between different types of HCP. Community health volunteers scored lower on most questions than other HCP. This can be attributed to the fact that community health volunteers usually receive less medical education. It suggests that these volunteers may benefit from more extensive childhood cancer training than other HCP. The community health volunteers are the eyes of HCP in communities and can educate the general public and facilitate the referral process. Previous studies analyzing the efficacy of digital learning, such as our project, also show a positive impact [43].

The radio campaign in Bungoma County has reached a large audience based upon the network of the local radio stations with over 3,000,000 listeners. Additionally, the live question-and-answer session resulted in several phone calls to the radio station showing interest of the general public. The actual impact of radio messages however is hard to measure yet. Analysis of future referrals to MTRH on whether or not the families had heard the radio messages on childhood cancer will demonstrate the extent of its effectiveness. The majority of reported awareness programs worldwide focus solely on HCP training [12]. Few other childhood cancer campaigns focused on reaching the general public [44–47]. None of these campaigns mentioned the use of radio to target a bigger audience.

### Strengths and Limitations

To our knowledge, this study is the first to implement and evaluate a blended learning strategy for childhood cancer awareness in LMIC. When implemented appropriately, this strategy could benefit many HCP in LMIC, also on diseases beyond childhood cancer. Limitations of the study include that due to this comprehensive multi-component strategy it was challenging to measure the exact impact of each component separately. Moreover, only one county in Kenya was included and that baseline knowledge level of HCP prior to the live training session was not measured. Moreover, participants of the SMS learning faced numerous technical challenges in accessing the successive elements of training material. This led to exclusion of a large group of HCP. Future research should include long-term follow-up to accurately measure impact on referrals and outcomes of children with cancer.

## Conclusion

This study described the implementation of a childhood cancer awareness program in western Kenya engaging HCPs and the general public. The program improved HCP’s knowledge of childhood cancer and increased the number of referrals of children with cancer. The project underlines that raising awareness can be a crucial tool in reducing underdiagnosis of children with cancer in Kenya and LMIC in general. Implementing awareness campaigns can ensure timely access to care and ultimately improve survival chances for children with cancer in LMIC.

## Supplementary Information

Below is the link to the electronic supplementary material.

Supplementary file1 (DOCX 15 KB)

## Data Availability

Data can be made available upon reasonable request.

## Abbreviations

LMIC: Low- and middle-income country
HIC: High-income country
HCP: Healthcare provider
NHIF: National Health Insurance Fund
SMS: Short message service
