# Supplementary material for: Childhood Cancer Awareness Program in Bungoma County, Kenya
Source: J Cancer Educ. 2024 Jun 22;40(1):54–64. doi: 10.1007/s13187-024-02468-z (PMC11846767; doi:10.1007/s13187-024-02468-z)
Supplement: Supplementary file 1 — Supplementary file1 (DOCX 15 KB) [file 13187_2024_2468_MOESM1_ESM.docx]

**Supplementary File 1.** Content of radio messages

| **Radio message topic 1: basic information on what childhood cancer is**  Childhood cancer occurs in children below 18 years of age. Every year approximately 400.000 children get cancer every year. Child cancer is curable. It is rare but important to recognise, as it is curable when caught early and adequate treatment with chemotherapy and sometimes surgery or radiotherapy is started. In some communities perceptions of community members upon cancer or childhood cancer specifically can differ. Child cancer is not a curse. However, in communities childhood cancer is often looked upon as a curse and therefore guardians sometimes seek for care in Traditional, Complementary and Alternative Medicine (TCAM) such as traditional healers and witch doctors. However childhood cancer cannot be cured by traditional healers or witch doctors, it can only be treated and then cured with chemotherapy and in some cases surgery. Childhood cancer is a non- communicable disease, just like for example hypertension or diabetes. Childhood cancer is sometimes confused to be a type of infection and to be transmittable. This is not the case, childhood cancer is not transmittable. |
| --- |
| **Radio message topic 2: when to think of childhood cancer and what to do when you suspect cancer**  There are different types of cancer. Cancer can occur anywhere in the body. It can occur in the blood, any organ and even in the brain. The two most important subtypes of cancer are blood cancers and solid cancers. Blood cancers usually present with persistent hotness of the body (fevers), pallor, bleeding, bruises, bonepains and recurrent infections. It is easy to confuse this with for example malaria or any other infection. Solid cancers usually present as a growth at the affected site. It is important to think of cancer when a child has a hard feeling mass that is not painful. Also when a child has persistent pallor, fevers (hotness of body) and bleeding or bruises. When a child has a cancer in the brain it is even more important to recognize this early. The child would then present symptoms such as convulsions, morning headache, morning vomiting and gait disturbances. Also problems with hearing and vision can occur. Sometimes the child can have balance problems or behavioral changes. Also for this type of cancer, traditional healers and witch doctors can not treat it. |
| **Radio message topic 3: NHIF coverage**  When you suspect your child has cancer it is important to go to a nearby health facility. The HCP can then assess the symptoms and if necessary refer to Moi Teaching and Referral Hospital (MTRH).  NHIF covers treatment costs for children with cancer.  Hospital detention practices are common in many healthcare facilities in LMICs. When a child is registered with NHIF hospital detention policies will not be applicable. The monthly NHIF contribution covers all the cancer treatment. NHIF costs on average 500 Kenyan Shilling per month. This insurance covers the whole family. The registration process usually takes up to one month. During the registration process a waiver can be used to cover initial treatment costs. |

**Supplementary File 2.** Characteristics of participants who completed pre-test, online SMS-course and post-test (n=890)

|  | **Total** | **Level 2 health facility** | **Level 3 health facility** |
| --- | --- | --- | --- |
| **Facilities**  **All participants**  **Type of staff**  Nurse  Community health volunteer  Community health extension worker  Public health officer  Clinical officer  Lab technician  Support staff* | 144  890 (100%)    39 (4%)  765 (85%)  32 (4%)  11 (1)  6 (0.5%)  4 (0.5%)  33 (4%) | 125 (87%)  774 (87%)  31 (4%)  668 (86%)  25 (3%)  9 (1%)  4 (0.5%)  4 (0.5%)  30 (4%) | 19 (13%)  116 (13%)  8 (7%)  98 (85%)  3 (3%)  2 (2%)  2 (2%)  0 (0%)  3 (3%) |

*Support staff included: clerks, casuals, mentor mothers, patient educators, students, health records and information officers, HIV testing services officers, and nutritionists.
